# Depression and Quality of Life Among Caregivers of Pediatric Cancer Patients

**DOI:** 10.7759/cureus.24256

**Published:** 2022-04-18

**Authors:** Meshal Alaqeel, Fahad Alkhathaami, Abdulelah Alshangiti, Abdullah Alanazi, Meshal A Alothri, Alwaleed T Alqarni, Fawaz I Almahmoud, Emad Masuadi

**Affiliations:** 1 Department of Psychiatry, Ministry of National Guard - Health Affairs, Riyadh, SAU; 2 Research Office, King Abdullah International Medical Research Center, Riyadh, SAU; 3 College of Medicine, King Saud Bin Abdulaziz University for Health Sciences, Riyadh, SAU; 4 Research Unit/Biostatistics, King Saud Bin Abdulaziz University for Health Sciences/King Abdullah International Medical Research Center (KAIMRC), Riyadh, SAU

**Keywords:** supportive and palliative care, quality of life, pediatric cancer, depression, caregivers

## Abstract

Background

This study aimed to assess the prevalence of depression, depressive symptoms, and quality of life among caregivers of pediatric cancer patients and the associated risk factors.

Methodology

In total, 73 participants were recruited for this cross-sectional study in King Abdullah Specialist Children Hospital. Two self-administered questionnaires were used, the Patient Health Questionnaire 9 (PHQ9) and World Health Organization Quality of Life (WHOQOL), to assess the depressive symptoms as well as the prevalence of clinical depression and quality of life, respectively. Data were analyzed using SPSS (IBM Corp., Armonk, NY, USA) to assess the level of depression and quality of life and the associated factors using Fisher’s exact and Mann-Whitney tests.

Results

It was found that 90.4% were females and 9.6% were males, with 49.3% being between the ages of 31 and 40. Regarding the level of depression, 47.80% had mild depression. There were no significant associations between the baseline characteristics and the level of depression. Gender was significantly associated with all four domains of quality of life, age was significant in physical health and environmental domains, duration of illness was significant only in the physical health domain, while education level was found to be not significantly associated with any of the domains.

Conclusions

This study found that nearly half of the participants had mild levels of depression, and the four domains of the WHOQOL were significantly affected by several risk factors. We recommend further research into this topic with larger sample sizes, as well as a follow-up assessment of caregivers for a more accurate representation of caregivers’ depression and quality of life. We recommend that in addition to the assessment of pediatric cancer patients, caregivers must also be assessed due to the burden associated with the task of being a caregiver.

## Introduction

Due to the long-term nature of cancer treatment, the primary care environment has transitioned from being in the hospital to being within a patient’s home, with family members functioning as primary caregivers [1]. Therefore, a cancer diagnosis not just affects the patient but also impacts the entire family physically, financially, and mentally [1-3].

Caregivers of cancer patients have been reported to experience significant grief and sadness, leading to mental stress and increasing the risk of developing psychiatric diseases [4]. Indeed, caregivers of cancer patients have been reported to experience depression, anxiety, social phobias, and mood changes [4,5].

Psychological disturbances are highly prevalent in the general population, and research has shown that depression levels are even higher in caregivers of pediatric cancer patients. Previous meta-analyses have shown that gender is an important predictor of this distress [6,7], with other studies reporting significant associations between psychological distress and being young, female, and employed. However, these findings have not been reported consistently across studies. Moreover, the rate of distress has been reported to be higher among caregivers of pediatric cancer patients who have increased supportive care needs, caregiver load, poorer quality of life, and lack of support [6-8]. According to research, the prevalence of depression among caregivers is 42.30%, while the prevalence of anxiety is 46.56% [4].

In addition to the previously mentioned psychological burdens faced by caregivers, they have an increased risk of physical illnesses, such as stroke, coronary artery disease, and fatigue [9,10]. Research has shown that caregiving is associated with higher five-year mortality risk (63%) in elderly individuals between 66 and 99 years of age [10].

Many caregivers of cancer patients also suffer from an increased financial burden, including a loss of productive hours due to care provision. Research has shown that caregivers dedicate an average of 22-40 hours per week to caregiving. Additional costs can result from out-of-pocket expenses, including special foods, parking, travel to appointments, medication, formal care, and extra household costs to support the patient [11].

Between 1999 and 2008, childhood cancers accounted for approximately 8% of all cancer cases in Saudi Arabia. The most commonly encountered pediatric cancers were leukemia (34.1%), lymphoma (15.2%), brain cancer (12.4%), and kidney cancer (5.3%). The overall incidence of childhood cancers increased from 8.8/100,000 in 1999 to 9.8/100,000 in 2008, and the incidence rates of cancers per 100,000 were generally higher in males than in females in 1999 and 2008 (9.4 and 11.5 in males vs. 8.3 and 8.1 in females, respectively). The highest incidence rates were in the birth to the four-year-old group during the surveyed years [12].

Studies have been conducted on primary caregivers, including fathers, mothers, brothers, and sisters, who care for pediatric cancer patients within their own homes, with many studies concentrating on early-stage cancer or not specifying a specific stage. However, the results of these studies were obtained using different designs, instruments, and methods, making it difficult to compare them.

To our knowledge, there have been no related studies conducted in Saudi Arabia with a focus on caregivers of pediatric cancer patients admitted to a pediatric oncology department without a specific disease stage. Therefore, this study aimed to assess the prevalence of depressive symptoms and the quality of life in caregivers of this population of pediatric cancer patients.

## Materials and methods

Study design, area, and settings

A descriptive, cross-sectional study was conducted in the pediatric oncology outpatient clinics and inpatient wards of our institution. This study involved two self-administered questionnaires completed by caregivers of pediatric cancer patients who met the inclusion criteria and expressed verbal consent.

Study participants

Study participants were Saudi caregivers of pediatric cancer patients who were aged 18 years and older and could read and write the Arabic language. Participants who could not complete the questionnaires were excluded from the study. A total of 73 consecutive participants were enrolled in this study, although 370 total participants were required to achieve a 95% confidence interval (CI) and 5% margin of error based on the Raosoft sample calculator [13].

Data collection process

Two questionnaires, the Patient Health Questionnaire 9 (PHQ-9) and the World Health Organization Quality of Life (WHOQOL) assessment, were distributed to caregivers of pediatric cancer patients in the pediatric oncology clinics and wards of our institution. Both questionnaires have been translated into Arabic and validated [14,15]. Baseline characteristics of participants were also collected, including age, gender, education level, and duration of child’s illness.

The WHOQOL is used to assess the quality of life of an individual over the past four weeks in four domains, including physical health (Domain 1), psychological health (Domain 2), social relationships (Domain 3), and environmental health (Domain 4). This questionnaire includes 30 items, with 26 items having scores ranging from to 1-5, and each score assigned a meaning depending on the question. Questions 27-30 are used to calculate the score of each domain based on specific equations.

The PHQ-9 questionnaire assesses a variety of depression-associated symptoms experienced over the past two weeks, including anhedonia, disturbed sleep, change in energy levels, etc. This questionnaire includes a total of nine items, with scores of 0-3 assigned based on the frequency of symptoms, including not at all (0), several days (1), more than half the days (2), and nearly every day (3). A collective score of 0-4 indicates no sign of depression, 5-9 indicates mild depression, 10-14 indicates moderate depression, 15-19 indicates moderately severe depression, and 20-27 indicates severe depression. While the diagnosis of major depressive disorder could be made if the following criteria were met, five items or more are checked as more than half the days (2), and either item (a) or (b) should be checked with at least more than half the days (2).

Data analysis

All data were entered into an Excel sheet and then transferred to SPSS version 23.0 (IBM Corp., Armonk, NY, USA). Categorical variables are reported as frequencies with percentages, and numerical variables are reported as means and standard deviation (SD). P-values were calculated using Mann-Whitney, Kruskal-Wallis, and Fisher’s exact tests.

## Results

In total, 73 caregivers participated in this study, including seven (9.6%) males and 66 (90.4%) females, with the majority aged between 31 and 40 years (49.3%). The educational levels attained by caregivers were 19.2% with less than high school, 43.8% with high school, and 37.0% with college degrees. In total, 33 (47.80%) caregivers were found to have mild levels of depression according to the PHQ-9 questionnaire. Only eight (10.95%) of the total participants were diagnosed with major depressive disorder. Table 1 shows the baseline characteristics of the participating caregivers, while Table 2 shows the descriptive characteristics of the four domains of the WHOQOL questionnaire.

**Table 1:** Baseline characteristics.

Gender	Male	7	9.6%
	Female	66	90.4%
Age (years)	≤30	19	26.0%
	31–40	36	49.3%
	41+	18	24.7%
Education level	Less than high school	14	19.2%
	High school	32	43.8%
	College	27	37.0%
Duration of illness	Up to 6 months	16	21.9%
	6–12 months	23	31.5%
	1–3 years	21	28.8%
	More than 3 years	13	17.8%
Depressive symptoms	Minimal	14	20.30%
	Mild	33	47.80%
	Moderate	14	20.30%
	Moderately severe	4	5.8%
	Severe	4	5.8%
Depression	Yes	8	11.0%
	No	65	89.0%

**Table 2:** Descriptive statistics of the four domains of the WHOQOL. SD: standard deviation; WHOQOL: World Health Organization Quality of Life

Descriptive statistics				
	Minimum	Maximum	Mean	SD
D1_100	25	100	63.3	17.2
D2_100	25	100	58.3	16.4
D3_100	0	100	63.5	19.9
D4_100	31	100	65.1	15.5

Our analysis found no significant associations between these baseline characteristics and the level of depression in caregivers, with a p-value of <0.05. Table 3 shows the frequency of these characteristics based on the level of depression with respective P-values. However, we were able to find a significant association between the duration of the disease and the diagnosis of major depressive disorder (p = 0.049). No other baseline characteristic was shown to have a significant association (Table 4).

**Table 3:** The association between baseline characteristics and PHQ-9 results with relative P-values. PHQ-9: Patient Health Questionnaire 9

		Patient Health Questionnaire								P-value
		Minimal		Mild		Moderate		Moderately severe/Severe		
		N	%	N	%	N	%	N	%	
Gender	Male	1	14.3%	4	57.1%	2	28.6%	0	0.0%	0.839
	Female	13	21.0%	29	46.8%	12	19.4%	8	12.9%	
Age (years)	≤30	4	23.5%	8	47.1%	2	11.8%	3	17.6%	0.087
	31–40	3	8.6%	19	54.3%	8	22.9%	5	14.3%	
	41+	7	41.2%	6	35.3%	4	23.5%	0	0.0%	
Education	Less than high school	4	30.8%	4	30.8%	3	23.1%	2	15.4%	0.425
	High school	4	13.3%	14	46.7%	7	23.3%	5	16.7%	
	College	6	23.1%	15	57.7%	4	15.4%	1	3.8%	
Duration of illness	Up to 6 months	4	30.8%	6	46.2%	2	23.1%	0	0.0%	0.06
	6–12 months	1	4.3%	9	39.1%	7	30.4%	6	26.1%	
	1–3 years	4	20.0%	13	65.0%	2	10.0%	1	5.0%	
	More than 3 years	5	38.5%	5	38.5%	2	15.4%	1	7.7%	

**Table 4:** The association between baseline characteristics and diagnosis of major depressive disorder results with relative p-values.

Depression		Depression				P-value
		No		Yes		
		N	%	N	%	
Gender	Male	7	100.0%	0	0.0%	1
	Female	58	87.9%	8	12.1%	
Age (years)	≤30	16	84.2%	3	15.8%	0.238
	31–40	31	86.1%	5	13.9%	
	41+	18	100.0%	0	0.0%	
Education	Less than high school	12	85.7%	2	14.3%	0.265
	High school	27	84.4%	5	15.6%	
	College	26	96.3%	1	3.7%	
Duration of illness	Up to 6 months	16	100.0%	0	0.0%	0.049
	6–12 months	17	73.9%	6	26.1%	
	1–3 years	20	95.2%	1	4.8%	
	More than 3 years	12	92.3%	1	7.7%	

The associations between baseline characteristics and the four domains of the WHOQOL are shown in Tables 4-7 along with p-values. For Domain 1, the mean score was 63.3 ± 17.2, and significant associations were found with gender (p = 0.021), age (p = 0.037), and duration of child’s illness (p = 0.048) (Table 5). For Domain 2, the mean score was 58.3 ± 16.4, and a significant association was found only with gender (p = 0.008) (Table 6). For Domain 3, the mean score was 63.5 ± 19.9, and a significant association was only found with gender (p = 0.012) (Table 7). Finally, for Domain 4, the mean score was 65.1 ± 15.5, and significant associations were found with gender (p = 0.02) and age (p = 0.009) (Table 8).

**Table 5:** The association between baseline characteristics and Domain 1 (physical QOL) results with relative p-values. SD: standard deviation; QOL: quality of life

		D1_100					P-value
		Mean	SD	Median	Q1	Q3	
Gender	Male	77.9	15.3	81	63	94	0.021
	Female	61.8	16.7	63	50	69	
Age (years)	≤30	69.8	15	69	56	81	0.037
	31–40	58.1	18	56	47	72	
	41+	66.8	14.9	63	56	81	
Education	Less than high school	62.6	21.1	56	50	88	0.887
	High school	6.32	19.3	63	50	78	
	College	6.38	12.2	63	56	69	
Duration of illness	Up to 6 months	72	14.4	69	63	81	0.048
	6–12 months	59.3	15.5	56	50	69	
	1–3 years	65.5	15.2	63	56	81	
	More than 3 years	56	22	50	44	69	

**Table 6:** The association between baseline characteristics and Domain 2 (psychological QOL) results with relative p-values. SD: standard deviation; QOL: quality of life

		D2_100					P-value
		Mean	SD	Median	Q1	Q3	
Gender	Male	74.3	12.9	69	69	88	0.008
	Female	56.6	15.9	56	44	69	
Age (years)	≤30	63.6	15.2	69	50	75	0.222
	31–40	55.5	16.6	56	44	66	
	41+	58.4	16.9	56	44	69	
Education	Less than high school	58.2	17.8	50	44	75	0.217
	High school	55.8	18.3	50	44	69	
	College	61.4	13.1	56	56	69	
Duration of illness	Up to 6 months	62.6	20.1	59.5	50	78	0.102
	6–12 months	53.7	15.8	50	44	63	
	1–3 years	62.9	14.1	63	56	75	
	More than 3 years	53.9	14.2	56	44	63	

**Table 7:** The association between baseline characteristics and Domain 3 (social QOL) results with relative p-values. SD: standard deviation; QOL: quality of life

		D3_100					P-value
		Mean	SD	Median	Q1	Q3	
Gender	Male	84	20	94	75	100	0.012
	Female	61.3	18.7	56	50	75	
Age (years)	≤30	69.1	21.4	69	50	94	0.324
	31–40	59	19.8	56	50	75	
	41+	66.7	17	72	50	75	
Education	Less than high school	69.2	17.1	72	50	81	0.508
	High school	62.3	22.2	56	47	78	
	College	62	18.4	56	50	75	
Duration of illness	Up to 6 months	67.6	25.9	72	53	81	0.356
	6–12 months	59.9	19.8	50	50	69	
	1–3 years	64.8	17.8	56	50	81	
	More than 3 years	62.9	15.2	69	50	75	

**Table 8:** The association between baseline characteristics and Domain 4 (environmental QOL) results with relative p-values. SD: standard deviation; QOL: quality of life

		D4_100					P-value
		Mean	SD	Median	Q1	Q3	
Gender	Male	77.9	14.5	75	75	88	0.02
	Female	63.7	15.1	63	50	69	
Age (years)	≤30	71.5	13.8	75	56	81	0.009
	31–40	60	15.2	56	50	69	
	41+	68.4	15	69	56	75	
Education	Less than high school	66.6	16	62.5	56	75	0.052
	High school	61	15.6	56	50	75	
	College	69.1	14.4	69	63	75	
Duration of illness	Up to 6 months	65.8	19.3	59.5	53	81.5	0.263
	6–12 months	62	16.1	56	50	75	
	1–3 years	70.1	13.1	69	63	75	
	More than 3 years	61.6	11.9	63	50	69	

## Discussion

We believe this study enhances our understanding of the factors that can increase the risk of depression in caregivers of pediatric cancer patients. In total, 73 caregivers of pediatric cancer patients were included in this study. Using the PHQ-9, we found that 47.80% of participants exhibited mild depressive symptoms without identification of any significant predictors for the level of depression. On the other hand, the WHOQOL questionnaire, which evaluates four domains of quality of life, exhibited significant associations for each domain.

In this study, we found that 14 (20.30%) participants had minimal symptoms of depression, 33 (47.80%) had mild symptoms, 14 (20.30%) had moderate symptoms, four (5.8%) had moderately severe symptoms, and four (5.8%) had severe symptoms of depression. Only eight (10.95%) participants, all of whom were female, were diagnosed with major depressive disorder, the only baseline characteristic affecting the diagnosis was duration of illness, with those with a duration of 6-12 months being the majority of those diagnosed [6]. Yeoh et al. [16] assessed depressive symptoms and their predictors in 728 participants from Malaysia. They found that 465 (63.9%) patients had minimal levels of depression, 110 (15.1%) had mild, 80 (11.0%) had moderate, and 73 (10%) had severe levels of depression. However, this study used the Beck Depression Inventory-II [17] which differs from the PHQ-9 used in our study. In addition, their sample size was larger and representative of the general population, rather than focusing exclusively on caregivers of pediatric cancer patients.

Similarly, a study conducted in southeastern Saudi Arabia screened 272 family medicine patients for depression using the PHQ-9 questionnaire, finding that 55% of patients had mild depression. Moreover, the diagnosis of clinical depression was exhibited in 12% of the participants which is consistent with our findings [18]. In a screening study of the general population of Saudi Arabia with a sample size of 477, 50.1% of patients exhibited minimal levels of depression using the PHQ-9. In contrast, this study showed that nearly half of the caregivers exhibited mild levels of depression. Indeed, it has previously been suggested that caring for a child with cancer can increase the probability that a caregiver will develop mild or more severe levels of depression [19]. In a study conducted in South India, depression was diagnosed in caregivers of patients diagnosed with breast cancer using the International Classification of Disease 10, with severity assessed using the Hamilton Depression Rating Scale. In that study, most caregivers had depression (n = 202, 52.5%), with most having mild depression (35%) [20].

In this study, the four domains of the WHOQOL were used to assess the quality of life of caregivers of pediatric cancer patients. Gender was the only significant factor for all four domains. In addition, age was a significant factor for the environmental and physical health domains, and the duration of illness was a significant factor for the physical health domain. A similar cross-sectional study was conducted in a general adult population from Iran. The study assessed the quality of life and depression levels in caregivers (n = 63) of patients with breast cancer. In that study, the authors analyzed correlations between depression and quality of life, with depression rates of 17.5% in participants with a lower quality of life, 42.9% in participants with a moderate quality of life, and 38.7% in participants with a higher quality of life. The authors found negative correlations between depression rates and lower income and education levels [21]. A related Chinese study assessed the quality of life of family caregivers of elderly individuals with chronic diseases using the 36-item Short Form Survey, which focuses on physical and mental health, with results showing poorer overall mental health and better physical health in a larger sample size (n = 407) [22]. Moreover, another study found a high correlation between age and poorer quality of life, which was associated with a number of predictors, including chronic illnesses, sleep disturbances, memory problems, anxiety, loneliness, and a sedentary lifestyle. However, in that study, the sample size was small (n = 55) with participants aged 60-90 years, whereas the present study included adult caregivers of all ages [23].

This study had some limitations. First, the study was limited by its cross-sectional design. Second, due to circumstances beyond our control in the form of the coronavirus disease 2019 pandemic, we were restricted in our ability to distribute our questionnaires and limited our sample size compared to our desired sample size and other literature in the field. Third, follow-up assessments of participants’ depression levels and quality of life could not be performed. Fourth, other factors that may have contributed to depression levels could not be assessed. Finally, this study was conducted in a single center.

## Conclusions

Caregivers of pediatric cancer patients need to provide care to their children throughout the day, which can limit the amount of time they can devote to meeting their own needs. In this study, we examined how the quality of life of these caregivers can be compromised. We found that nearly half of the participants exhibited mild depressive symptoms. We also found that six to twelve months is when caregivers are at the most risk to develop major depressive disorders; hence, they should be screened during that time. There were no significant associations between baseline characteristics and levels of depression; however, there were significant associations between some baseline characteristics and the four domains of quality of life. For example, caregivers of higher age or female gender should have more focused assessments during visits. In future studies, we will recruit more participants to reach the required sample size. In addition, we recommend follow-up assessments of caregivers to understand their depression levels and quality of life more accurately. Based on this study, we recommend regular assessments of caregiver needs due to the intense burdens associated with caring for pediatric cancer patients.
